# Alternative chromatographic system for the quality control of lipophilic technetium-99m radiopharmaceuticals such as [^99m^Tc(MIBI)_6_]^+^

**DOI:** 10.1590/1414-431X20144489

**Published:** 2015-03-03

**Authors:** D.P. Faria, C.A. Buchpiguel, F.L.N. Marques

**Affiliations:** 1 Centro Translacional de Oncologia, Instituto do Câncer do Estado de São Paulo, São Paulo, SP, Brasil; 2 Serviço de Medicina Nuclear, Departamento de Radiologia, Faculdade de Medicina, Universidade de São Paulo, São Paulo, SP, Brasil

**Keywords:** Radiopharmaceuticals, Technetium-99m, Quality control, Chromatography

## Abstract

Knowledge of the radiochemical purity of radiopharmaceuticals is mandatory and can be evaluated by several methods and techniques. Planar chromatography is the technique normally employed in nuclear medicine since it is simple, rapid and usually of low cost. There is no standard system for the chromatographic technique, but price, separation efficiency and short time for execution must be considered. We have studied an alternative system using common chromatographic stationary phase and alcohol or alcohol:chloroform mixtures as the mobile phase, using the lipophilic radiopharmaceutical [^99m^Tc(MIBI)_6_]^+^ as a model. Whatman 1 modified phase paper and absolute ethanol, Whatman 1 paper and methanol:chloroform (25:75), Whatman 3MM paper and ethanol:chloroform (25:75), and the more expensive ITLC-SG and 1-propanol:chloroform (10:90) were suitable systems for the direct determination of radiochemical purity of [^99m^Tc(MIBI)_6_]^+^ since impurities such as^99m^Tc-reduced-hydrolyzed (RH),^99m^TcO_4_^-^ and [^99m^Tc(cysteine)_2_]^-^complex were completely separated from the radiopharmaceutical, which moved toward the front of chromatographic systems while impurities were retained at the origin. The time required for analysis was 4 to 15 min, which is appropriate for nuclear medicine routines.

## Introduction

Radiopharmaceutical quality control is mandatory for manufacturers prior to human use. Nowadays, compounded technetium-99m radiopharmaceuticals need to be evaluated after their preparation in the nuclear medicine department to avoid injection of improper radiochemical purity (RCP) and to attend legal requirements. The system most frequently used for the control of technetium-99m radiopharmaceuticals is planar chromatography (1) since it is usually less expensive (2), is efficient in separating compounds from impurities, and requires less time for analysis. Several quality control procedures use two or three chromatographic systems to permit the desired identification of radiopharmaceuticals and regular impurities, such as unreacted pertechnetate-99m (^99m^TcO_4_^-^), reduced-hydrolyzed technetium-99m (^99m^TcRH) and, in specific cases, the ^99m^Tc-primary complex. However, their use may increase cost and time for analysis; thus, a simple system is more desirable than multiple systems.

The radiopharmaceutical [^99m^Tc(MIBI)_6_]^+^ is a well-known cationic and hydrophobic compound used for myocardial perfusion imaging during routine diagnostic procedures or during acute myocardial infarction (3). It has been used as a successful diagnostic agent for the detection of breast and lung tumors (4). To obtain accurate clinical information with [^99m^Tc(MIBI)_6_]^+^, manufacturers recommend an RCP higher than 90% (5,6). It is well known that a low RCP has been obtained when employing procedures not recommended by the manufacturer, such as the use of high activity for labeling due to an eluate with low specific activity (7) or the use of fractionated cold kits (8).

The instruction for [^99m^Tc(MIBI)_6_]^+^quality control, described in the package insert for labeling Cardiolite^¯^ (5), is a time-consuming procedure due to the slow migration of 95% ethanol in the Al_2_O_3_ Baker-Flex strip. Other alternative and faster procedures described elsewhere are: solvent extraction using chloroform/saline (6); reverse phase mini-column chromatography (Sep-Pak-C18) (9); thin layer or partition chromatography using different systems such as instant thin layer chromatography-silica gel (ITLC-SG) and acetone and saline as the independent mobile phase (10); Gelman Solvent Saturation Pads and chloroform:tetrahydrofuran (11); and Whatman 31ET paper with ethyl acetate as mobile phase (12). However, the latter one was re-evaluated by another research group (13) and the radiochemical purity value was higher than when using the standard procedure (Al_2_O_3_ Baker-Flex strip/ethanol), giving a false-positive purity value.

The aim of the present investigation was to evaluate new chromatographic systems using chloroform (CHCl_3_) and different alcohols as the mobile phase, and Whatman paper or ITLC-SG as the stationary phase, looking for a fast, reproducible and low cost system for the analysis of RCP of [^99m^Tc(MIBI)_6_]^+^.

## Material and Methods

### Radiochemical species

Sodium [^99m^Tc]pertechnetate (Na^99m^TcO_4_) was eluted from a^99^Mo/^99m^Tc generator (IPEN-TEC) and used for labeling and as a standard for chromatographic analyses. [^99m^Tc(MIBI)_6_]^+^ was obtained using an in-house prepared lyophilized kit (6) containing the addition of 1.11-1.85 GBq of^99m^TcO_4_^-^ in 2 mL of saline solution, heated in boiling water for 10 min, and then allowed to reach room temperature.

Radiochemical impurities such as [^99m^Tc(cysteine)_2_]^-^ primary complex were obtained by an in-house preparation containing 1.0 mg L-cysteine hydrochloride, 2.6 mg sodium citrate, 20 mg mannitol, 0.075 mg SnCl_2_.2H_2_O, and labeled with 1.11-1.48 GBq of^99m^TcO_4_^-^ in 2 mL of saline solution, without heating. Hydrolyzed-reduced [^99m^Tc]technetium was obtained by the reaction of^99m^TcO_4_^-^ and 0.075 mg of SnCl_2_.2H_2_O in 2 mL of water solution.

### Chromatographic systems

Analytical grade methanol (MeOH), anhydrous ethanol (EtOH), 1-propanol (PrOH), 1-butanol (BuOH) and chloroform (CHCl_3_) were used without further purification, either pure or in alcohol:CHCl_3_ mixtures (10:90, 75:25, 50:50, 25:75). Whatman 1 paper (W1), Whatman 3MM paper (W3MM) and instant thin layer chromatography-silica gel (ITLC-SG) (Gelman Science, Inc., USA) were used as support or normal stationary phase. A modified stationary phase was prepared by washing W1 paper with anhydrous ethanol and drying in an oven at 80°C for 5 min.

### Chromatographic analysis

One drop of each sample was spotted 1 cm from the origin of the chromatographic support strips (1×8 cm), developed with solvent migrating 6 cm and immediately dried using hot air. The strips were cut into 6 parts of 1 cm each and radioactivity was measured using an NaI(Tl) well counter. The results are reported in terms of migration distance (cm) or retardation factor (Rf), calculated by dividing the distance traveled by the radioactive material by the distance traveled by the mobile phase.

### Data analysis

Data are reported as means±SD for more than three samples. The level of significance was set at P<0.05 (Student's*t*-test).

## Results

**Figure 1:**
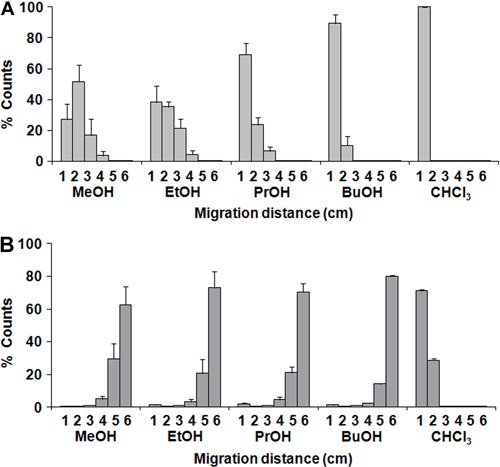
Paper chromatography of ^99m^TcO_4_^-^ (*A*) and [^99m^Tc(MIBI)_6_]^+^(*B*) reported as percent of counts in segments of the chromatographic strip (cm), numbers 1 to 6. Alcohols (MeOH: methanol, EtOH: ethanol, PrOH: 1-propanol, BuOH: 1-butanol) or chloroform (CHCl_3_) were used as the mobile phase, and Whatman 1 (W1) paper as the stationary phase [a similar behavior was obtained with Whatman 3MM (W3MM) paper, data not shown]. Data are reported as means±SD for n=3.

**Figure 2:**
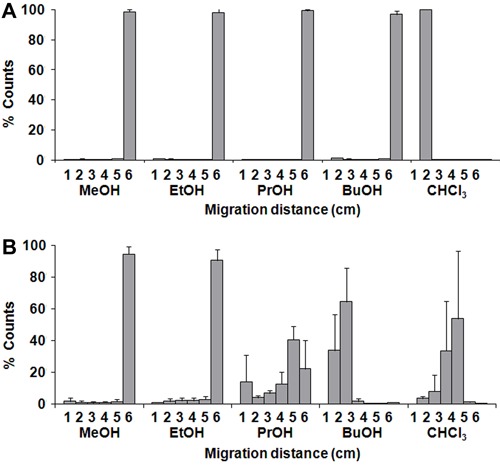
Thin layer chromatography of ^99m^TcO_4_^-^ (*A*) and [^99m^Tc(MIBI)_6_]^+^(*B*) reported as percent of counts in segments of the chromatographic strip (cm), numbers 1 to 6. Alcohols (MeOH: methanol, EtOH: ethanol, PrOH: 1-propanol, BuOH: 1-butanol) or chloroform (CHCl_3_) were used as the mobile phase, and ITLC-SG as the stationary phase. Data are reported as means±SD for n=3.

Chromatographic analyses using alcohol or CHCl_3_ for the different radiolabeled compounds showed that ^99m^Tc-RH and the [^99m^Tc(cysteine)_2_]^-^ primary complex were retained in the Rf=0.0-0.16 (origin), independent of the stationary or mobile phase. For [^99m^Tc(MIBI)_6_]^+^and^99m^TcO_4_^-^, different behaviors were observed with the different systems. Using W1 or W3MM papers as stationary phases and CHCl_3_ as the mobile phase, both species remained at the origin. On the other hand, when different alcohols were used [^99m^Tc(MIBI)_6_]^+^ moved toward the Rf=0.66-1.0 (front) regardless of the alcohol used, while^99m^TcO_4_^-^ was retained at Rf=0.00-0.16, especially using PrOH or BuOH, as shown in Figure 1. Using ITLC-SG as the stationary phase and CHCl_3_, [^99m^Tc(MIBI)_6_]^+^ was found at Rf=0.33-0.66, while ^99m^TcO_4_^-^ remained at the origin. For the same stationary phase, but using alcohols, ^99m^TcO_4_^-^ was found at Rf=0.66-1.0 independent of the alcohol used, while most of the [^99m^Tc(MIBI)_6_]^+^ was retained at the origin, but mobility increased with the decreasing length of alcohol carbon chain, as demonstrated in Figure 2.

The ITLC-SG stationary phase had a faster running time compared to the W1 and W3MM. Furthermore, running time increased with increasing alcohol carbon chain length, requiring 10 min in the W1 paper/methanol system and 35 min in W1 paper/BuOH, but the fastest system was that using CHCl_3_ as the mobile phase, as shown inTable 1.

**Table:**
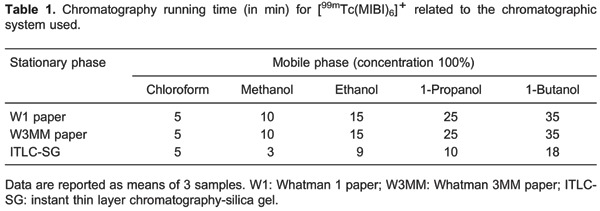

Since the effects reported were for individual solvents, we evaluated different alcohol:CHCl_3_ mixtures. The best results were obtained with the mixtures whose compositions are presented in Table 2. These systems allow efficient separation of [^99m^Tc(MIBI)_6_]^+^, with Rf=0.66-1.00, from other possible radiochemical species such as^99m^TcO_4_^-^, ^99m^Tc-RH and the ^99m^Tc-primary complex, which had Rf=0.0-0.16, allowing the use of a single strip to determine RCP, as shown inFigure 3.

**Figure 3:**
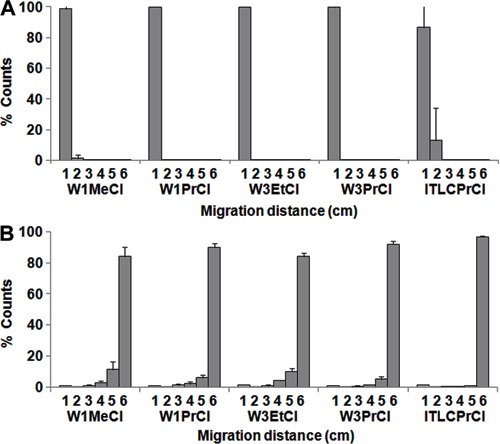
Chromatography of ^99m^TcO_4_^-^ (*A*) and [^99m^Tc(MIBI)_6_]^+^(*B*) reported as percent of counts in segments of the chromatographic strip (cm), numbers 1 to 6. The following chromatographic systems were used: (W1MeCl) W1 paper/methanol:chloroform (25:75), (W3EtCl) W3 paper/ethanol:chloroform (25:75), (W1PrCl) W1 paper/propanol:chloroform (25:75), (W3PrCl) W3 paper/propanol:chloroform (25:75), and (ITLCPrCl) ITLC-SG/propanol:chloroform (25:75). Data are reported as means±SD for n=18.

**Table:**
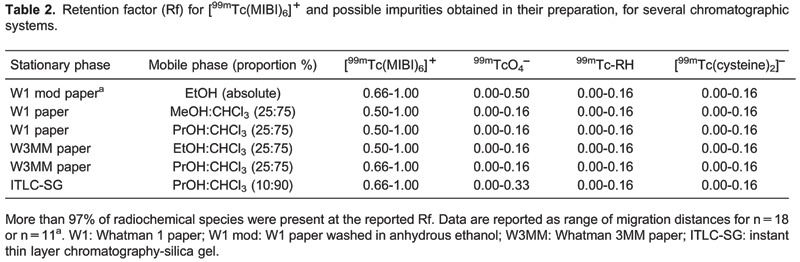

Chromatographic stationary phases can be modified by washing or by covering it with chemical reactants. In the present study, W1 paper was washed with anhydrous ethanol, and the behavior of the radiochemical species changed slightly, showing less dispersion of [^99m^Tc(MIBI)_6_]^+^in relation to the front and of ^99m^TcO_4_^-^ in relation to the origin compared to normal W1 paper, as presented inFigure 4. This paper was kept in a closed container and was used up to 15 days after preparation.

**Figure 4:**
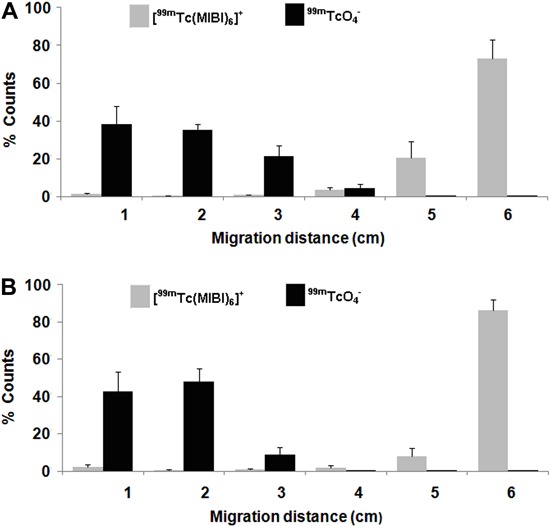
Paper chromatography of ^99m^TcO_4_^-^ and [^99m^Tc(MIBI)_6_]^+^ in Whatman 1 (W1) regular paper (*A*) and W1 modified paper (*B*) reported as percent of counts in segments of the chromatographic strip (cm), numbers 1 to 6. Anhydrous ethanol was used as the mobile phase. W1 modified paper is W1 paper washed with anhydrous ethanol. Data are reported as means±SD for n=18 (*A*) or n=11 (*B*).

The time required for chromatographic analyses in the modified stationary phase and mixed mobile phase are shown in Table 3. The best result was obtained for the ITLC-SG/PrOH:CHCl_3_ system, which had times between 4 and 5 min.

**Table:**
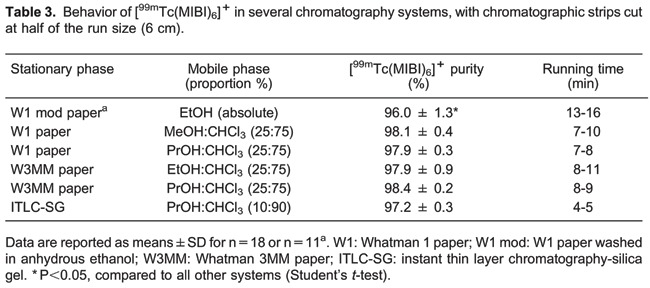

An important issue is that, except for the W1 modified paper/EtOH system, no statistical difference (Student's *t*-test, P>0.05) was observed for radiochemical purity value comparing all the used systems.

## Discussion

Planar chromatography is an analytical method, which depends on two separation mechanisms (14,15). One is based on the process of adsorption of the product to the stationary phase, such as Al_2_O_3_ or silica-gel, and desorption of the molecules by the mobile phase or solvent. The second mechanism involves the partition of radiochemical species between the immiscible solvent and the water binding or adsorbed to the cellulose fibers of the paper support. These two processes have been used for [^99m^Tc(MIBI)_6_]^+^quality control, with adsorption phenomena observed for the Al_2_O_3_/EtOH systems (5) and the double system ITLC-SG/saline and ITLC-SG/acetone (10). Partition chromatography can be observed in the Whatman 31ET paper/ethyl acetate systems (12), in which [^99m^Tc(MIBI)_6_]^+^ was extracted by hydrophobic solvents, allowing it to migrate away from the origin of the chromatographic system.

Based on the literature (5,11,12), we decided to evaluate the use of CHCl_3_, instead of ethyl acetate to extract [^99m^Tc(MIBI)_6_]^+^ from the aqueous phase of the paper chromatography. Unexpectedly, the radiopharmaceutical was retained at the origin of the chromatographic system together with other radiochemical species, independent of the use of W1 or W3MM paper, as shown in Figure 1B. As both ethyl acetate and CHCl_3_ are hydrophobic, these divergent results could be explained by the low solubility (16) of CHCl_3_in water (0.08 mL in 10 mL) when compared with ethyl acetate (1 mL in 10 mL), which hinders the formation of the water/CHCl_3_ binary phase necessary for the extraction of [^99m^Tc(MIBI)_6_]^+^ into the organic phase.

To evaluate the effect of mobile phase solubility in water bound to the cellulose fibers of W1 and W3MM papers during the separation of radioactive impurities and [^99m^Tc(MIBI)_6_]^+^, we used alcohols such as methanol, ethanol and 1-propanol, all completely soluble in water, and 1-butanol (0.9/10 mL), whose water solubility is similar to that of ethyl acetate (1/10 mL) (16). Impurities such as^99m^Tc-RH and ^99m^Tc-cysteine were retained at the origin (Rf=0.0-0.16) in all of the systems used. The behavior observed for^99m^TcO_4_^-^ (Figure 1A) is associated with the hydrophilic characteristic of ^99m^TcO_4_^-^, which is soluble in water and in several other polar protic solvents such as alcohols. During chromatographic separation, alcohols solvate^99m^TcO_4_^-^, transporting it from the origin to other regions in the stationary phase, but the process is affected by carbon chain length, as observed for water-soluble 1-propanol, which is less effective for the solvation of the inorganic anion. The behavior observed for 1-butanol is associated with low solubility in water, with a decreased ability to extract compounds from the water phase. In contrast, [^99m^Tc(MIBI)_6_]^+^ is a hydrophobic compound, with 6 methoxy groups able to perform hydrogen bonding to alcohol, which is solvated and transported during mobile phase evolution. Although [^99m^Tc(MIBI)_6_]^+^ is highly soluble in CHCl_3_, the low solubility of this mobile phase in water does not allow the extraction of the radiopharmaceutical (Figure 1B).

On the basis of these results, 1-butanol would be the choice for mobile phase, but the 35-min time needed to develop the chromatograph (Table 1) is too long for nuclear medicine routines.

Chromatographic separation in silica-gel (ITLC-SG) is proposed to occur by an adsorption/desorption mechanism. Although ^99m^TcO_4_^-^ is presented as an anionic molecule, it is a resonance hybrid and its interaction with the stationary phase is weak when compared to a polar mobile phase consisting of alcohols, which results in displacement of the compound from the origin of the chromatographic system to the solvent front. On the other hand, although CHCl_3_ is considered to be a polar mobile phase, it is less polar than alcohol, and did not displace ^99m^TcO_4_^-^ from the sample application point (Figure 2A). The behavior of [^99m^Tc(MIBI)_6_]^+^ cannot be explained by a single interaction or separation mechanism because of the specific characteristics of the molecule: 1) it is a cationic compound, 2) it has high lipophilic characteristic with logP=1.1 (17), and 3) it has 6 methoxy groups able to form hydrogen bonding. For these reasons, the interactions of the analyte with the stationary and mobile phases are complex (Figure 2B). For the ITLC-SG system, only with the use of 1-butanol was it possible to efficiently separate^99m^TcO_4_^-^ from [^99m^Tc(MIBI)_6_]^+^(Figure 2), but with the system showing a relatively long run time (Table 2) and it was not considered for use routine use.

Our results indicated that mixtures of CHCl_3_ and alcohols could be used to reach a rapid and efficient separation of the radiochemical species discussed here. Among the several mixtures evaluated by us for RCP, the best results were obtained with alcohol:CHCl_3_ (25:75) (Table 2 and Figure 3). Under these conditions, for paper chromatography, it was possible to form a ternary solvent system consisting of water:alcohol:CHCl_3_, allowing [^99m^Tc(MIBI)_6_]^+^ to be extracted by water bound to cellulose (Figure 3B), while^99m^TcO_4_^-^ was dissolved in the water (Figure 3A). For the ITLC-SG system, the best results were obtained when the PrOH:CHCl_3_ (10:90) mixture was used. This contrasts with the results obtained for paper chromatography using MeOH:CHCl_3_, EtOH:CHCl_3_and PrOH:CHCl_3_ (25:75).

Other mixtures of different proportions were evaluated but their behavior regarding separation efficiency, reproducibility and time did not satisfy the requirements of an efficient quality control procedure. The final results for the BuOH:CHCl_3_ (25:75) mixture were excluded because they did not show an advantage over other alcohols.

Although the present results are acceptable, several nuclear medicine services are not equipped with a fume hood that would permit to work safely with CHCl_3_. Thus, we investigated an alternative chromatographic system by modifying the paper water phase, washing it with ethanol to allow the change of water bound to the cellulose surface by ethanol, as a way to modify interactions between analyte, mobile phase (ethanol) and stationary phase. With this procedure, the migration rate of the ^99m^TcO_4_^-^ was decreased, concentrating it close to the origin, while the migration of the [^99m^Tc(MIBI)_6_]^+^increased, allowing the best separation between the species (Figure 4A and 4B). Although this last system has shown RCP value statistically different from the systems using alcohol:CHCl_3_mixtures (Table 3), in practice, the numerical difference was small and the RCP results obtained could be considered comparable to those observed for other systems, as value differences were around 1-3%, and this could be within technical error (large sample spotted, millimetric difference on sample spot position or on the paper cutting position, or solvent saturation degree into the chromatographic chamber).

In conclusion, the chromatographic systems investigated showed high separation efficiency of [^99m^Tc(MIBI)_6_]^+^ from other possible radiochemical species, as shown by analysis of the possible impurities. The systems provided a rapid and reproducible alternative method for the determination of the radiochemical purity of [^99m^Tc(MIBI)_6_]^+^, when compared with standard procedure (Al_2_O_3_ Baker-Flex strip/95% ethanol) that takes about 20 min to be performed and the degree of alcohol hydration is important for reproducibility. Furthermore, the results point to the use of these systems to determine the radiochemical purity of other technetium lipophilic radiotracers or radiopharmaceuticals.

## References

[1] Decristoforo C, Zolle I, Rakiás F, Imre J, Hesslewood SR, Zolle I (2007). Quality control methods of ^99m^Tc
pharmaceuticals. Technetium-99m pharmaceuticals. preparation and quality control
in nuclear medicine.

[2] Lima MJC, Marques FLN, Okamoto MRY, Garcez AT, Sapienza MT, Buchpiguel CA (2005). Preparation and evaluation of modified composition for
lyophilized kits of [Cu(MIBI)_4_]BF_4_ for [^99m^Tc]
technetium labeling. Braz Arch Biol Technol.

[3] Bauer A, Mehilli J, Barthel P, Muller A, Kastrati A, Ulm K (2009). Impact of myocardial salvage assessed by
(99m)Tc-sestamibi scintigraphy on cardiac autonomic function in patients
undergoing mechanical reperfusion therapy for acute myocardial
infarction. JACC Cardiovasc Imaging.

[4] Mohan HK, Miles KA (2009). Cost-effectiveness of ^99m^Tc-sestamibi in
predicting response to chemotherapy in patients with lung cancer: systematic
review and meta-analysis. J Nucl Med.

[5] Anonymous (1991). Cardiolite [package insert H-23534].

[6] Faria DP, Marques FLN, Yamada AS, Miquelin CA (2011). Evaluation of costs for quality control of
[^99m^Tc]technetium radiopharmaceuticals in Brazilian nuclear medicine
centers. Radiol Bras.

[7] Hung JC, Herold TJ, Gibbons RJ (1996). Optimal conditions of ^99m^Tc eluate for the
radiolabeling of ^99m^Tc-sestamibi. Nucl Med Biol.

[8] Varelis P, Parkes SL, Poot MT (1998). The influence of generator eluate on the radiochemical
purity of ^99^Tc^m^-sestamibi prepared using fractionated
Cardiolite kits. Nucl Med Commun.

[9] Reilly RM, So M, Polihronis J, Houle S (1992). Rapid quality control
of^99^Tc^m^-sestamibi. Nucl Med Commun.

[10] Proulx A, Ballinger JR, Gulenchyn KY (1989). Routine determination of radiochemical purity
of^99m^Tc-MIBI. Int J Rad Appl Instrum A.

[11] Hung JC, Wilson ME, Brown ML, Gibbons RJ (1991). Rapid preparation and quality control method for
technetium-99m-2-methoxy isobutyl isonitrile
(technetium-99m-sestamibi). J Nucl Med.

[12] Zimmer AM, Spies SM (1991). Quality control procedures for newer
radiopharmaceuticals. J Nucl Med Technol.

[13] Luebke AL, Wilary DM, Mahoney DW, Hung JC (2000). Evaluation of an alternative radiochemical purity
testing method for technetium-99m sestamibi. J Nucl Med Technol.

[14] Braithwaite A, Smith FJ, Braithwaite A, Smith FJ (1996). Planar chromatography. Chromatographyc methods.

[15] Ravindranath B (1989). Principles and practice of chromatography.

[16] American Chemical Society - Division of Organic Chemistry Common organic solvents: Table of
properties. http://www.organicdivision.org/orig/organic_solventes.html.

[17] Zhou Y, Liu S (2011). ^64^Cu-labeled phosphonium cations as PET radiotracers for tumor
imaging. Bioconjug Chem.

